# Therapeutic effects of hydrogen on chronic graft‐versus‐host disease

**DOI:** 10.1111/jcmm.13155

**Published:** 2017-04-04

**Authors:** Liren Qian, Xiaopeng Liu, Jianliang Shen, Defeng Zhao, Wenjie Yin

**Affiliations:** ^1^ Department of Hematology Navy General Hospital Beijing China; ^2^ Seamen/Aircrew Physical Examination Center Navy General Hospital Beijing China

**Keywords:** Chronic graft‐versus‐host disease, stem cell transplantation, hydrogen, treatment

## Abstract

The incidence of chronic graft‐versus‐host disease (cGVHD) is rising recent years, which has been the leading cause of non‐transplantation mortality post allogenetic hematopoietic stem cell transplantation (HSCT). Imbalance of inflammatory cytokines and fibrosis plays critical roles in the pathogenesis of cGVHD. Recent studies showed that molecular hydrogen has anti‐inflammatory, antioxidant, anti‐fibrosis effects. Therefore, we hypothesized that molecular hydrogen may have therapeutic effects on cGVHD. To determine whether hydrogen could protect mice from cGVHD in an MHC‐incompatible murine bone marrow transplantation (BMT) model, survival rates of mice were calculated, and skin lesions were also evaluated after BMT. This article demonstrated that administration of hydrogen‐rich saline increased survival rate of cGVHD mice. Administration of hydrogen‐rich saline after transplantation also reduced skin lesions of cGVHD mice. Previously, we reported the therapeutic effects of hydrogen on acute GVHD. However, there was no report on the therapeutic effects of hydrogen on cGVHD mice. It is suggested that hydrogen has a potential as an effective and safe therapeutic agent on cGVHD. This study will provide new ideas on the treatment of cGVHD and has important theoretical values.

## Background

Allogeneic hematopoietic stem cell transplantation (allo‐HSCT) has been widely used in benign and malignant haematological diseases. With the wide use of peripheral blood stem cells as a graft, the increase application of allo‐HSCT in elderly patients, and improvement in the early survival rate after transplantation, the incidence of cGVHD is rising year by year 1, which has become the leading cause of non‐transplantation related death 2.

The exact mechanism of cGVHD is still unclear. But it is widely accepted that inflammatory factors imbalance and fibrosis occupy the dominant position in the development of cGVHD 3. In 2007, Ohsawa *et al*. 4 discovered that hydrogen gas has antioxidant and anti‐apoptotic properties. Since then, hydrogen gas has come to the forefront of therapeutic medical gas research. Recent basic and clinical researches 5, 6, 7, 8 proved that hydrogen could down‐regulate cytokines, including CCL2, IL‐1β, IL‐6, IL‐12, TNF‐α, etc. In 2011, Terasaki *et al*. also demonstrated that hydrogen has anti‐fibrosis effect 9. Since 2009, it was demonstrated that hydrogen could protect allograft function in intestinal transplantation, lung transplantation, renal transplantation and heart transplantation models 10, 11, 12, 13, 14, 15. We also reported the therapeutic effects of hydrogen gas on acute graft‐versus‐host disease (aGVHD) 16, 17 after allo‐HSCT. Because of the anti‐inflammatory and anti‐fibrosis effects, we hypothesized that hydrogen may have therapeutic effects on cGVHD.

In this study, we investigated whether administration of hydrogen‐rich saline exerted therapeutic effects on cGVHD mice. We demonstrated here that hydrogen treatment could increase survival rate of cGVHD mice and improve skin lesions of cGVHD mice.

## Materials and methods

### Hydrogen‐rich saline production

As we previously reported 18, 19, 20, hydrogen was dissolved in physiological saline 6 hrs under high pressure (0.4 MPa) to a supersaturated level using hydrogen‐rich water producing apparatus produced by our department. The saturated hydrogen saline was stored under atmospheric pressure at 4°C in an aluminium bag with no dead volume. Hydrogen‐rich saline was freshly prepared every week, which ensured that a concentration of more than 0.6 mM was maintained. Gas chromatography (Biogas Analyzer Systems‐1000, Mitleben, Japan) was used to confirm the content of hydrogen in saline by the method described by Ohsawa *et al*. 4.

### Mice

All the protocols were approved by the Navy General Hospital, China in accordance with the Guide for Care and Use of Laboratory Animals published by the US NIH (publication No. 96‐01). Recombination activating gene two‐targeted (RAG‐2KO) mice on the BALB/c background and B10.D2 mice were obtained from Jackson Laboratories (Bar Harbor, ME, USA). All mice were studied at between 6 and 10 weeks of age. Mice were housed in autoclaved cages with sterile food and water.

### cGVHD model

cGVHD model was established as described by Ruzek *et al*. 21. Spleens were harvested aseptically from B10.D2 mice, and the tissue was dissociated by rubbing, between sterile frosted glass slides, into RPMI 1640 medium containing 10% foetal calf serum (FCS), 1% L‐glutamine and 1% penicillin/streptomycin solution (complete medium) (all reagents from Gibco BRL, Grand Island, NY, USA) to generate a single‐cell suspension. Red blood cells were lysed by 1‐2‐minute incubation with 150 mM ammonium chloride lysis solution (Sigma‐Aldrich, St. Louis, MO, USA), viable cells were enumerated by trypan blue dye exclusion on a hemocytometer, and the cells were resuspended in RPMI 1640 containing 10 units/ml heparin (Sigma‐Aldrich). Prior to injection, cells were filtered through a 75‐μm nylon mesh cell strainer (Becton Dickinson, Franklin Lakes, NJ, USA) to remove large debris. Between 2 × 10^7^ and 5 × 10^7^ B10.D2 (for induction of GVH), spleen cells were injected intravenously into recipient BALB/c RAG‐2 KO mice. Mice were treated intraperitoneally (IP) with physiological saline or hydrogen‐rich saline (5 ml/kg) 20 days after transplantation every day.

### Survival assays

To evaluate therapeutic effects of hydrogen, mice were returned to the animal facility and routinely cared for 60 days after transplantation. Survival was checked and scored daily for 60 days.

### Evaluation of cGVHD

Following treatment, animals were scored for skin manifestations of cGVHD every 5 days. The following scoring system was used as follows: healthy appearance equals 0; skin lesions with alopecia less than 1 cm^2^ in area, 1; skin lesions with alopecia 1–2 cm^2^ in area, 2; skin lesions with alopecia more than 2 cm^2^ in area 3; Additionally, animals were assigned 0.3 point each for skin disease (lesions or scaling) on the ears, tail and paws. Minimum score was 0, maximum score 3.9. Incidence and clinical score curves represent all mice that achieved a score of 0.6 or higher. Final scores for humanely killed animals were kept in the data set for the remaining time points of the experiment.

### Tissue histopathology

Shaved skin from the interscapular region (approximately 2 cm^2^) was fixed in 10% formalin, embedded in paraffin, sectioned, slide mounted and stained with haematoxylin and eosin. Slides were scored by a dermatopathologist blinded to experimental groups on the basis of dermal fibrosis, fat loss, inflammation, epidermal interface changes and follicular drop‐out (0–2 for each category). Minimum score was 0, maximum score 10. HE‐stained skin preparations of sclerodermatous skin lesions were assessed at 55 day after transplantation. Pathologic cGVHD involvement of the skin was independently assessed on a scale from 0 to 8 for each mouse.

### Statistical analysis

Survival curves were constructed using the Kaplan–Meier product limit estimator and compared using the log‐rank rest. Other results are expressed as mean ± S.D. and compared by the two‐sample Student's *t*‐test for differences in means. *P* < 0.05 was deemed to be significant in all experiments.

## Results

### Hydrogen increased survival rate of cGVHD mice

**Figure 1:**
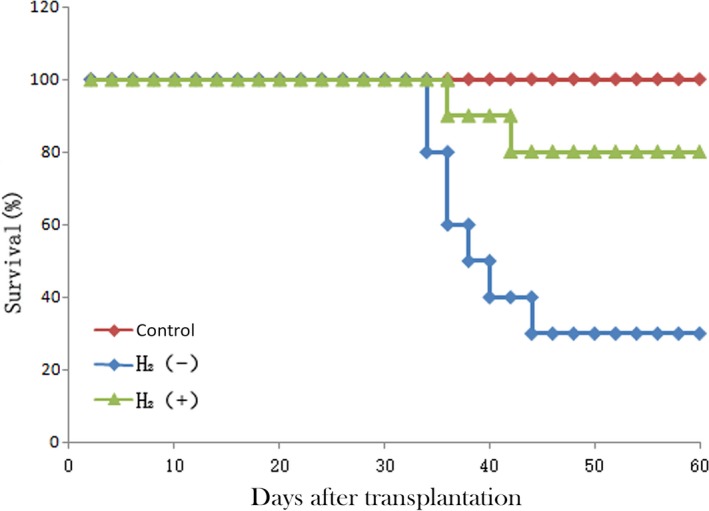
Administration of hydrogen‐rich saline intraperitoneally from the 20th day after transplantation protects mice from cGVHD (*n* = 30, *P* < 0.05).

Initial studies were performed to determine whether hydrogen could protect mice from cGVHD in a murine model. Mice were treated IP with physiological saline or hydrogen‐rich saline (5 ml/kg) 20 days after transplantation every day. 70% of cGVHD mice without H_2_ treatment died by the 60th day after transplantation (Fig. 1, *P* < 0.05), while 80% of the mice pre‐treated with H_2_ survived (Fig. 1). Thus, H_2_ may have therapeutic effects on cGVHD.

### Hydrogen improves skin lesions of cGVHD mice

**Figure 2:**
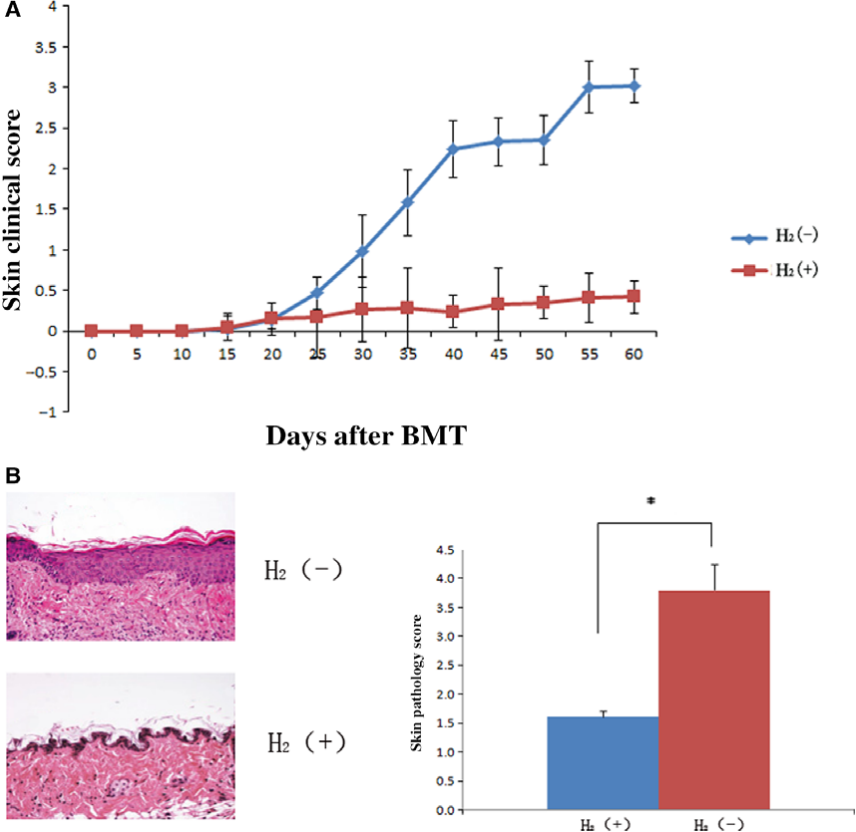
Hydrogen‐rich saline was administered intraperitoneally every day from 20th day after transplantation. (**A**) Skin clinical scores were evaluated every 5 days. (**B**) HE‐stained skin preparations of sclerodermatous skin lesions were assessed at 55 day after transplantation. Pathologic cGVHD involvement of the skin was independently assessed on a scale from 0 to 8 for each mouse. Cohort averages are displayed. (*n* = 30, *P < 0.05. Error bars indicate S.E.M.).

To determine the therapeutic effects of hydrogen, the skin clinical scores were evaluated every 5 days and HE‐stained skin preparations of sclerodermatous skin lesions were assessed at 55th day after transplantation. Pathologic cGVHD involvement of the skin was independently assessed on a scale from 0 to 8 for each mouse. As shown in Figure 2A, the skin symptoms were improved, and clinical scores were significantly decreased by hydrogen (*P* < 0.05). At 55th day after transplantation, pathologic skin score was 1.6 in the hydrogen group which was significantly less than 3.8 in the control group (Fig. 2B, *P* < 0.05).

## Discussion

Although the incidence of cGVHD is rising year by year, and cGVHD has become the most intractable complication after allo‐HSCT, there is still no ideal therapeutic method on the disease. In the past three decades, glucocorticoids, calcineurin inhibitors (*e.g*. tacrolimus and cyclosporin) and other immunosuppressive agents are still the main drugs for cGVHD. The course of cGVHD is often more than 3 months. Long‐time use of glucocorticoids and other immunosuppressive agents often accompany with severe side effects include severe infection, ulcer, femoral head necrosis, osteoporosis, weight gain, diabetes, high blood pressure, emotional instability, etc. The side effects of these drugs are always too severe to be tolerated. However, hydrogen has few side effects which can be used for long time safely. It is continuously produced by colonic bacteria in the body and normally circulates in the blood 22. Inhalation of hydrogen gas does not influence physiological parameters such as body temperature, blood pressure, pH and pO_2_ in the blood 4, 6. It is physiologically safe for humans to inhale hydrogen. This feature makes hydrogen can be used for long time on cGVHD.

In this study, we demonstrated that hydrogen treatment could increase the survival rate of cGVHD mice and improve skin lesions of cGVHD mice. Recently, we have reported a patient with cGVHD successfully treated with hydrogen‐rich water 23. To our knowledge, this is the first study demonstrating that hydrogen has therapeutic effect on cGVHD mice. The mechanism may rely on the anti‐inflammatory, antioxidant and anti‐fibrosis ability of hydrogen. However, the exact mechanism is still not clear. However, the exact mechanism and the signalling pathway involved in the therapeutic role of hydrogen in cGVHD needs to be studied in the future.

## Funding

This study was supported by Innovative Cultivation Foundation of Chinese Navy General Hospital (Grant No.CXPY201603).

## Authors' contributions

Liren Qian and Xiaopeng Liu contribute equally to the paper. Liren Qian and Jianliang Shen designed the research. Defeng Zhao analysed the data and prepared the typescript. Xiaopeng Liu revised the manuscript. The other authors provided the subject data. All authors read and approved the final manuscript.

## Competing interests

The authors declare that they have no competing interests.

## Ethics approval and consent to participate

The study was approved by the Ethics Committee of Navy General Hospital, Beijing, China.
